# Quantitative analysis of speckle-based X-ray dark-field imaging using numerical wave-optics simulations

**DOI:** 10.1038/s41598-021-95227-9

**Published:** 2021-08-09

**Authors:** Sebastian Meyer, Serena Z. Shi, Nadav Shapira, Andrew D. A. Maidment, Peter B. Noël

**Affiliations:** 1 Department of Radiology, Perelman School of Medicine, University of Pennsylvania, Philadelphia, PA 19103 USA; 2 Department of Diagnostic and Interventional Radiology, School of Medicine and Klinikum rechts der Isar, Technical University of Munich, 81675 Munich, Germany

**Keywords:** X-rays, Imaging techniques, X-rays

## Abstract

The dark-field signal measures the small-angle scattering strength and provides complementary diagnostic information. This is of particular interest for lung imaging due to the pronounced small-angle scatter from the alveolar microstructure. However, most dark-field imaging techniques are relatively complex, dose-inefficient, and require sophisticated optics and highly coherent X-ray sources. Speckle-based imaging promises to overcome these limitations due to its simple and versatile setup, only requiring the addition of a random phase modulator to conventional X-ray equipment. We investigated quantitatively the influence of sample structure, setup geometry, and source energy on the dark-field signal in speckle-based X-ray imaging with wave-optics simulations for ensembles of micro-spheres. We show that the dark-field signal is accurately predicted via a model originally derived for grating interferometry when using the mean frequency of the speckle pattern power spectral density as the characteristic speckle size. The size directly reflects the correlation length of the diffuser surface and did not change with energy or propagation distance within the near-field. The dark-field signal had a distinct dependence on sample structure and setup geometry but was also affected by beam hardening-induced modifications of the visibility spectrum. This study quantitatively demonstrates the behavior of the dark-field signal in speckle-based X-ray imaging.

## Introduction

X-ray dark-field imaging measures small-angle X-ray scattering caused by the microstructure of granular materials^1^ on a scale substantially below the spatial resolution of the imaging system, which is not possible with conventional X-ray imaging. This has recently gained increased attention for diagnostic lung imaging since multiple refractions from alveolar tissue-air interfaces result in a pronounced dark-field signal. Structural variations of the pulmonary alveoli due to the progression of respiratory diseases are associated with a reduction of the dark-field signal compared to a healthy lung parenchyma. Several pre-clinical in vivo studies have demonstrated the potential of X-ray dark-field imaging for early stage diagnosis of lung disease such as fibrosis^2,3^, emphysema^4,5^ or pneumothorax^6^. Respiratory diseases in general are among the leading causes of severe illness and death worldwide^7^ and early detection plays a critical role in slowing disease progression and initiating effective treatments. Therefore, dark-field imaging has the potential to ease the immense health burden imposed by respiratory disease.

Since conventional X-ray detectors are only sensitive to X-ray intensity, specialized X-ray optics are necessary to convert phase effects into a measurable intensity difference. Various solutions for phase-sensitive imaging have been developed in the past 50 years^8^ including many that allow the inference of dark-field information, such as Talbot–Lau grating interferometry^1^, coded-aperture^9^ and analyzer-based techniques^10^, and (free-space) propagation-based imaging^11^. These approaches have different clinical potential due to their various requirements for spatial and temporal coherence, the fact that the hardware is complex and expensive, and the dose efficiency of the acquisitions. Despite substantial developments, many challenges for a broad clinical translation of phase-sensitive X-ray imaging still remain^12,13^.

**Figure 1:**
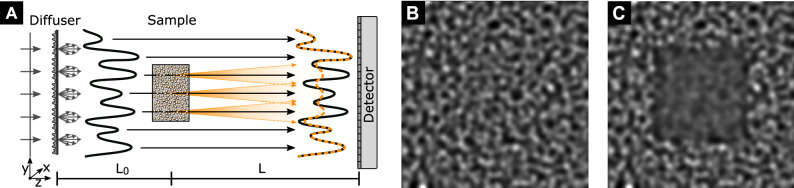
Principle of speckle-based dark-field imaging. (**A**) Coherent X-rays impinge on the diffuser, are randomly scattered, and mutually interfere with the incident beam to create a random intensity (speckle) pattern (black solid line), which does not change upon propagation within the near-field regime. Diffuse small-angle X-ray scattering from the sample microstructure causes a measurable visibility reduction of the original speckle pattern (orange dashed line), which corresponds to the dark-field signal. In this illustration, beam divergence, transmission and phase-contrast effects are neglected. Drawing is not to scale. (**B**) Simulated speckle pattern in the detector plane for a setup without sample and (**C**) for a cube filled with micro-spheres, resulting in a reduction of the speckle visibility.

Near-field X-ray speckle imaging^14^ has recently been proposed as a simple but versatile alternative for multi-contrast X-ray imaging, i.e., the simultaneous acquisition of transmission, phase-contrast, and dark-field signals. In addition, the coherence requirements of speckle imaging are only moderate^15,16^. Speckles are a well-known phenomenon in optics and refer to intensity modulations arising from mutual interference of radiation scattered from small randomly-distributed features^17^. Interestingly, the speckle pattern does not change in near-field regime^18^, allowing it to act as a wavefront marker. The principle of speckle-based dark-field imaging is illustrated in Fig. 1. Coherent X-rays shining on a random phase modulator (diffuser) form a speckle pattern that is directly observed with a detector system. Scattering from the spatially-unresolved random microstructure of a sample placed in the X-ray beam will locally distort the wavefront, thereby causing a measurable visibility (amplitude) reduction of the original speckle pattern. In addition, the mean intensity of the speckle pattern will be reduced due to absorption in the sample. Refraction caused by an extended phase-gradient in the sample will result in a lateral displacement of the speckle pattern if the length scale is above the detector pixel pitch.

To retrieve multi-contrast images, the changes in the speckle pattern between a reference (sample-free) and sample image are evaluated. Digital image correlation algorithms^19^ are used to track the spatial distortions of the speckle pattern induced by the presence of the sample. The potential of speckle-based imaging has been demonstrated at synchrotrons and laboratory micro-focus X-ray tubes with various diffusers^20^, such as fine sandpaper, filter membranes, or steel wool.

Several studies have examined the dark-field signal strength for grating interferometry^21,22^. However, most studies in speckle-based X-ray imaging are focused on the speckle tracking accuracy and sensitivity for phase-contrast signal extraction^19,23–25^. A dedicated quantitative investigation of the speckle-based dark-field imaging performance is lacking. This would support the further development of speckle-based dark-field imaging systems and is highly relevant for evaluating potential pre-clinical and clinical applications.

To address this need, we systematically evaluate the dependence of the dark-field signal in speckle-based X-ray imaging. Based on numerical wave-optics simulations that account for the physical interactions underlying the image formation process, the influence of different sample characteristics and imaging setup parameters on the resulting dark-field signal are quantified and compared to theoretical predictions.

## Methods

Numerical wave-optics simulations were used to model beam propagation through a speckle-based X-ray imaging system. In the following we outline the general simulation technique, the imaging system configuration, as well as the quantitative dark-field signal retrieval method.

### Wave-optics simulation

The evolution of an X-ray wave-field in the presence of a static, nonmagnetic, and sufficiently slowly spatially varying medium is described by scalar diffraction theory^26^. To simulate speckle imaging, we need to model the transport of the X-ray wave-field through the complete imaging system, which consists of the propagation between different system components and the disturbance of the wave-field upon encountering an object. The free-space propagation by a distance $\Delta _z$ along the *z*-axis for a monochromatic wave-field $\psi _\omega$ of angular frequency $\omega = c k$, where $k=2\pi / \lambda$ is the radiation wave-number corresponding to a wavelength $\lambda$ and *c* is the speed of light, was calculated by the angular spectrum method^27^:

1 $$\begin{aligned} \psi _\omega \left( x,y,z+\Delta _z \right) = {\mathscr {F}}^{-1}\exp \left[ i \Delta _z \sqrt{k^2 - k_x^2 - k_y^2}\right] {\mathscr {F}} \, \psi _\omega \left( x,y,z \right) , \end{aligned}$$

where ${\mathscr {F}}$ and ${\mathscr {F}}^{-1}$ denote the Fourier transform and its inverse, and $k_x$ and $k_y$ are the angular components of the wave-vector. Hence, to propagate the wave-field in free-space (e.g., from diffuser to sample), we have to multiply the unpropagated field in its Fourier decomposition with the Fresnel propagator in Fourier space (cf. Eq. (1)).

The phase and amplitude shifts imparted on a wave-field traversing the material of an object contained within a slab of space between $z=z_0$ and $z=z_1$ are calculated in the projection approximation^28^:

2 $$\begin{aligned} \psi _\omega \left( x,y,z_1 \right) \approx \exp \left[ -i k \int \limits _{z=z_0}^{z=z_1} \left[ \delta _\omega \left( x,y,z\right) -i\beta _\omega \left( x,y,z\right) \right] dz \right] \psi _\omega \left( x,y,z_0 \right) , \end{aligned}$$

with $\delta$ and $\beta$ being the components of the refractive index ($n=1-\delta +i\beta$) distribution of the object, which were computed for each material using the *xraylib* library^29^. In the projection approximation we treat any three-dimensional object as projection of its material’s complex refractive index along its thickness in propagation direction (z-axis). This neglects diffraction within the object volume. The wave-field at the exit surface of an object (e.g., diffuser or sample) is obtained by multiplying the incoming wave-field with the object’s complex transmission function, i.e., the exponential term on the right-hand side of Eq. (2).

For propagating the spherical wave emitted by a point source, the divergent beam geometry was transformed to an equivalent parallel beam by applying a coordinate system transformation following the Fresnel scaling theorem^30^. This transformation involves a continuous rescaling of the real-space coordinate system at each propagation step. The polychromatic source was expressed by incoherently superimposing simulations performed at wavelengths throughout the source spectrum. To account for the extended focal spot size, we blurred the speckle patterns using a Gaussian kernel with a full width at half maximum equal to the source size scaled according to the divergent beam geometry. In summary, the simulation process relies on an iterative use of Eq. (1) to propagate the wave-field from the exit of one object to the beginning of the next one (i.e., diffuser, sample or detector) and Eq. (2) to calculate the disturbed wave-field exiting an object.

### Simulated speckle-based X-ray imaging setup

The number of grid points in the simulation was set to $4096\times 4096$. The initial grid spacing was ${0.2}\,{\upmu \hbox {m}}$ at the position of the diffuser and the grid coordinate system is continuously rescaled according to the divergent-beam-to-plane-wave transformation^30^. Throughout this study we simulated a point source with a nominal ${10}\,\upmu \hbox {m}$ focal spot located at the coordinate system origin (i.e., z=0 m). An ideal monochromatic source of 30 keV was simulated first. Then, a polychromatic 45 kVp spectrum of an X-ray tube (tungsten target and 1mm aluminum filtration; 30.1 keV average energy) was simulated. The emission spectrum was generated via the external software *SPEKTR*^31^. The diffuser was modeled as sandpaper sheets, each consisting of a rough aluminum oxide surface and a ${200}\,{\upmu \hbox {m}}$ thick cellulose backing. The diffuser was located at z=1m with an isotropic surface structure that was generated with a numerical two-dimensional Fourier filtering method^32^ assuming a Gaussian power spectral density (PSD) with correlation length $l_0$:

3 $$\begin{aligned} PSD\left( r\right) = \sigma ^2 \pi l_0^2\exp \left[ -\left( \pi l_0 r \right) \right] , \end{aligned}$$

where *r* is the radial frequency and $\sigma$ defines the root-mean-square (RMS) of the diffuser surface height probability density function (PDF). The rough surface generated using the PSD from Eq. (3) defines the varying aluminum oxide thickness of the top part of the diffuser. The effect of the diffuser on an impinging wave-field is then calculated by inserting the diffuser model’s three-dimensional refractive index distribution into Eq. (2). The detector was located at z=3.0 m (unless noted otherwise) and a ${10}\,{\upmu \hbox {m}}$ pixel pitch and a point spread function of one pixel full width at half maximum (FWHM) were assumed. This system geometry is similar to experimental setups with microfocus X-ray tubes used by other researchers^33^. The simulated sample (z=1.2 m, unless noted otherwise) was a cuboid containing randomly positioned and non-overlapping mono-disperse spheres made from glass (SiO$_2$, $\rho ={2.00}\,{\hbox {g}/\hbox {cm}^{3}}$) or PMMA (C$_5$O$_2$H$_8$, $\rho ={1.18}\,{\hbox {g}/\hbox {cm}^{3}}$). The effect of the sample on the wave-field is calculated via Eq. (2). A close packing^34^ was generated if the packing fraction was beyond the random packing limit. However, we used a packing fraction of 0.05 for most of this work, since generating a dense packing is computationally expensive and spheres cannot be considered fully random. The packing fraction is adjusted by changing the total number of spheres placed within the cubic sample volume. Sample thickness, packing fraction, sphere diameter, as well as sample position were varied throughout this work to investigate their impact on the dark-field signal.

### Multi-model signal extraction

X-ray speckle imaging allows for the simultaneous extraction of transmission, (differential) phase-contrast and dark-field contrast information. Since the central part of the sample used in this work does not contain regions of extended phase-gradients, speckle displacement (phase-contrast) can be neglected. The transmission of the sample was calculated for each pixel from the ratio between the measured local mean intensities in the sample and the reference image. In our case, the dark-field signal is given for each pixel by the ratio of the speckle pattern visibility between the sample ($v_{{\text {sam}}}$) and the reference ($v_{{\text {ref}}}$) scan^20,35^ (cf. Fig. 1):

4 $$\begin{aligned} D\left( x,y\right) = \frac{v_{{\text {sam}}}\left( x,y\right) }{v_{{\text {ref}}}\left( x,y\right) }= \frac{\sigma _{{\text {sam}}}\left( x,y\right) }{T\left( x,y\right) ~\sigma _{{\text {ref}}}\left( x,y\right) }, \end{aligned}$$

where $\sigma _{{\text {sam}}}$ and $\sigma _{{\text {ref}}}$ are the corresponding locally-calculated intensity standard deviations and *T* is the sample transmission. An analysis window larger than the average speckle size was moved across the sample and reference image in steps of one pixel to locally calculate the transmission and dark-field signals in each pixel. For each sample configuration the mean transmission and dark-field are obtained from the central $20\times 20$ detector pixels and averaged over simulations performed with 10 different diffusers and samples.

## Results

### Influence of the speckle pattern

It has been demonstrated that the dark-field signal of a sample with thickness *t* can be described by an exponential law $D=\exp (-\mu _d \, t )$^21,36^, where the visibility extinction coefficient $\mu _d$ for the ensemble of spherical particles of diameter *d* and packing fraction *f* used in this work is given by^37^:

5 $$\begin{aligned} \mu _d = \frac{3 \pi ^2}{\lambda ^2} f |\Delta n|^2 d {\left\{ \begin{array}{ll} 1-\sqrt{1-\frac{\xi ^2}{d^2}}\left( 1+\frac{1}{2}\frac{\xi ^2}{d^2}\right) +\left( \frac{\xi ^2}{d^2}-\frac{1}{4}\frac{\xi ^4}{d^4}\right) \times \ln \left[ \frac{\frac{d}{\xi }+\sqrt{\frac{d^2}{\xi ^2}-1}}{\frac{d}{\xi }-\sqrt{\frac{d^2}{\xi ^2}-1}}\right] , &{}\quad d>\xi .\\ 1, &{}\quad d\le \xi . \end{array}\right. } \end{aligned}$$

where $\Delta n$ is the difference of the complex refractive index between the sphere material and the surrounding air. $\xi = \frac{\lambda L}{p}$ is the autocorrelation distance of the setup given by the sample-to-detector distance *L* and the X-ray wavelength $\lambda$. Equation (5) was originally derived for grating interferometers where *p* corresponds to the grating period (or the period of the analyzer grating in a Talbot–Lau interferometer^21^), defining the structure of the illumination pattern. To account for the divergent beam emitted by a point source, *L* and *p* have to be scaled by the geometric magnification.

Analogously for the case of speckle-based X-ray imaging, *p* is hypothesized to represent the speckle size. Since a diffuser will create a random rather than a periodic intensity distribution, speckles do not have a well-defined size and only a mean measure can be obtained based on the statistical properties of the pattern. The speckle size is typically defined via the autocorrelation function^38,39^ or the power spectrum^16,40^.

**Figure 2:**
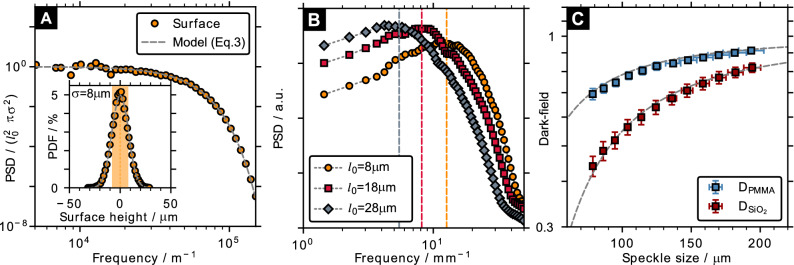
Influence of the speckle size on the dark-field signal. (**A**) Power spectral density (PSD) and probability density function (PDF) in comparison to the prescribed model of Eq. (3) for the surface structure of a single diffuser layer ($l_0 = {8}\,{\upmu \hbox {m}}$, $\sigma = {8}\,{\upmu \hbox {m}}$). (**B**) PSD of speckle patterns obtained for diffusers of different correlation lengths ($l_0$) and the corresponding mean frequencies (dashed lines). (**C**) Theoretical dark-field (**D**) signals (dashed lines) as a function of speckle size (*p*) according to Eq. (5) and corresponding simulation results for a cubic sample containing an ensemble of glass (SiO$_2$) or PMMA spheres ($f=0.05$, $t={5}\,{\hbox {mm}}$ and $d={4}\,{\upmu \hbox {m}}$) when using the mean frequency of the PSD as speckle size.

The azimuthally-averaged PSD and RMS surface-height PDF of a single diffuser surface are shown in Fig. 2A and are in excellent agreement with the expectations of the model (Eq. (3)). The corresponding PSD of the speckle pattern on the detector can be seen in Fig. 2B in comparison to the results obtained for sandpaper models of varying correlation length. The dashed lines indicate the average frequencies of the PSD, which correspond to speckle sizes of ${78.82}\,{\upmu \hbox {m}}$, ${122.76}\,{\upmu \hbox {m}}$ and ${183.18}\,{\upmu \hbox {m}}$ for $l_0 = {8}\,{\upmu \hbox {m}}$, $l_0 = {18}\,{\upmu \hbox {m}}$ and $l_0 = {28}\,{\upmu \hbox {m}}$, respectively. In contrast, the FWHM of the corresponding autocorrelation function yields values around 2.46 times smaller. Figure 2C shows the theoretical dependence of the dark-field signal (dashed lines) on *p* (i.e., the speckle size) according to Eq. (5) for a sample of $d={4}\,{\upmu \hbox {m}}$ glass and PMMA spheres. The simulated dark-field values are in good agreement with the theoretical model when using the speckle size defined by the average PSD frequency (Fig. 2B), meaning that it represents the correct characteristic length scale needed for a quantitative dark-field description. In the following, the simulated diffuser will consist of 5 sandpaper layers ($l_0 = {8}\,{\upmu \hbox {m}}$ and $\sigma = {8}\,{\upmu \hbox {m}}$), corresponding to an average speckle size of ${78.82}\,{\upmu \hbox {m}}$.

### Influence of sample parameters

**Figure 3:**
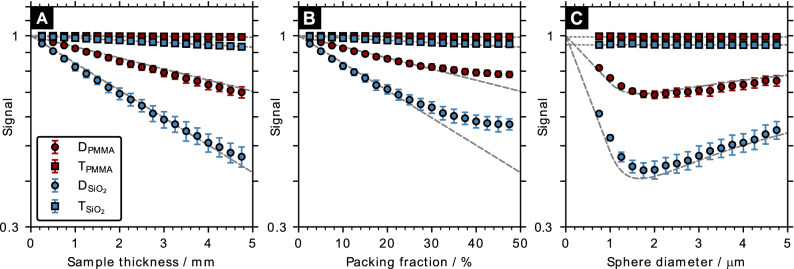
Influence of the sample properties on the dark-field signal. Semi-logarithmic plot of the dark-field (D) and transmission (T) signals’ dependence on sample thickness (**A**; $f=0.05$ and $d={4}\,{\upmu \hbox {m}}$), packing fraction (**B**; $t={0.5}\,{\hbox {mm}}$ and $d={4}\,{\upmu \hbox {m}}$) and sphere diameter (**C**; $f=0.05$ and $t={4}\,{\hbox {mm}}$) for a simulated cubic sample containing an ensemble of glass (SiO$_2$) or PMMA spheres. The dashed and dotted gray lines indicate the theoretical dark-field and transmission signals calculated according to Eqs. (5) and (6).

To validate the simulation results and highlight the characteristics of the dark-field signal, we also evaluated the transmission signal. The X-ray attenuation is given by the Beer–Lambert law and the theoretical transmission for a cuboid of thickness *t* filled with spheres of packing fraction *f* can be approximated by

6 $$\begin{aligned} T \approx \exp \left( - \mu f t\right) , \end{aligned}$$

where $\mu =2 k \beta$ is the linear attenuation coefficient of the sphere material. Figure 3 shows dark-field and transmission signals for different sample configurations. Both signals exhibit the expected exponential dependence on sample thickness and packing fraction (Fig. 3A,B). However, for large packing fractions (>30%) the dark-field exhibits a sub-linear trend. As shown in Fig. 3C, smaller spheres provide a stronger dark-field as more scattering structures are present in the sample until reaching a maximum sensitivity (i.e., lowest dark-field value) at $d \approx 1.8 \, \xi ={1.7}\,{\upmu \hbox {m}}$. The corresponding transmission signals do not vary due to the constant packing fraction. Overall, the calculated theoretical curves agree well with the simulation results.

### Influence of setup geometry

**Figure 4:**
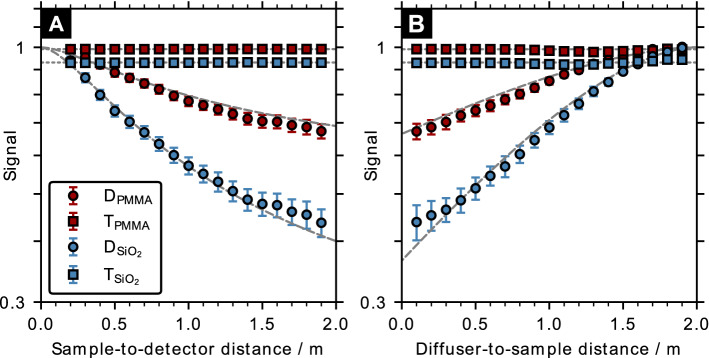
Influence of the setup geometry on the dark-field signal. Semi-logarithmic plot of the dark-field (D) and transmission (T) signals’ dependence on (**A**) sample-to-detector distance and (**B**) diffuser-to-sample distance for a cubic sample containing an ensemble of glass (SiO$_2$) or PMMA spheres ($f=0.05$, $t={5}\,{\hbox {mm}}$ and $d={4}\,{\upmu \hbox {m}}$). The dashed and dotted gray lines indicate the theoretical dark-field and transmission signals calculated according to Eqs. (5) and (6).

Figure 4 shows the impact of the setup geometry on the dark-field and transmission signals for a given sample configuration ($f=0.05$, $t={5}\,{\hbox {mm}}$ and $d={4}\,{\upmu \hbox {m}}$). Increasing the propagation distance by moving the detector further downstream resulted in a substantially lower dark-field value (Fig. 4A). The calculated theoretical dark-field signal implicitly assumes that the effective speckle size remains constant. Changing the sample position (Fig. 4B) while keeping the detector location fixed at z=3 m affects not only the propagation distance but also the speckle pattern at the sample due to geometric magnification. For the given setup, the dark-field signal exhibited an almost linear dependency (for a semi-log plot) for small diffuser-to-sample distances with minor deviations from theoretical predictions when the sample is placed extremely close to the diffuser. As in the previous investigation of the sample structure, the results match the theoretical predictions of Eq. (5) using the mean frequency of the speckle pattern PSD. In both scenarios the transmission signal was not affected, as expected from the Beer–Lambert law (Eq. (6)).

### Influence of source energy

**Figure 5:**
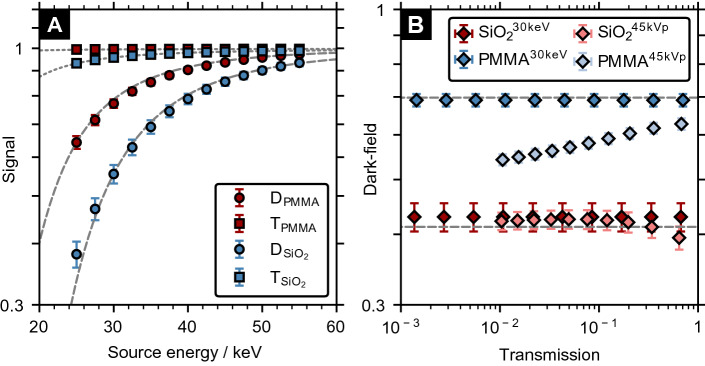
Influence of the source energy on the dark-field signal. (**A**) Semi-logarithmic plot of the dark-field (D) and transmission (T) signals’ dependence on the source energy for a cubic sample containing an ensemble of glass (SiO$_2$) or PMMA spheres ($f=0.05$, $t={3}\,\hbox {mm}$ and $d={3}\,{\upmu \hbox {m}}$). (**B**) Two-dimensional histogram plot showing the correlation of the dark-field and transmission signals for an increasing thickness (${0.01}\,\hbox {mm}$ to ${0.19}\,{\hbox {mm}}$) of lead between the diffuser and sample ($f=0.05$, $t={4}\,\hbox {mm}$ and $d={2}\,{\upmu \hbox {m}}$). A monochromatic 30 keV source (dashed gray lines indicate the theoretical dark-field signals calculated according to Eq. (5)) and a 45 kVp spectrum were considered.

The dependence of the dark-field and transmission signals on the source energy is shown in Fig. 5A. Both signals have similar non-linear energy dependence, which can be described by $\mu \propto E^{-3}$ and $\mu _D \propto E^{-3.2}$. The dark-field signal is in good agreement with the theoretical model assuming constant speckle size of ${78.82}\,{\upmu \hbox {m}}$ (i.e., the diffuser with $l_0={8}\,{\upmu \hbox {m}}$ and 30 keV). Figure 5B shows the effect of beam hardening on both signals when an increasing thickness (from 0.01 to ${0.19}\,{\hbox {mm}}$ in steps of ${0.02}\,{\hbox {mm}}$) of lead is placed between diffuser and sample. For a monoenergetic source the dark-field signal remained unchanged and increasing lead thickness resulted in a constant additive change (logarithmic scale) of transmission. For the 45 kVp spectrum (30.1 keV average energy before added filtration), beam hardening due to the lead layer causes an increasingly higher transmission compared to the monoenergetic case, as expected. The effect of beam-hardening on dark-field signal varied with the absolute dark-field signal strength. For a high dark-field signal (e.g., $D\approx 0.6$ for the sample with PMMA spheres), the dark-field signal decreases for decreasing transmission. By contrast, for the low dark-field signal ($D\approx 0.4$, for the sample with SiO$_2$ spheres), the initial dark-field reduction is much smaller. In addition, instead of further decreasing with decreasing transmission, the effect is reversed. For transmission below 0.1, the dark-field signal remained constant.

## Discussion

In this work, we quantitatively investigated the potential of speckle-based X-ray imaging for extracting the dark-field signal. Since the dark-field signal is related to a large number of scattering events from micro- and nanometer-sized structures, the dark-field contrast entails sub-resolution information. This has many important applications in medical imaging and industrial non-destructive material testing.

For diffusers typically used in X-ray speckle imaging, such as sandpaper, the dominant mechanism for speckle generation is the random interface between a surface and air. This means that the phase-shifts imparted to an incoming X-ray wave are primarily determined by the random surface-height fluctuations encountered^17^, making the presented diffuser model very useful for studies on the impact of the relevant diffuser parameters on the speckle pattern. Using the mean frequency of the PSD to define the average speckle size resulted in very good agreement between simulated and theoretical dark-field values. Hence, it represents the characteristic speckle length scale relevant for the dark-field signal strength, but the result may depend on the particular type of speckles generated. The mean frequency values were close to the mode frequencies, which was used by Zhou et al.^40^ to estimate the speckle size, but are less sensitive to fluctuations. The FWHM of the autocorrelation function^38,39^ provided systematically smaller speckle sizes and did not correctly predict the dark-field signal.

The deviation of the dark-field signal from the theoretical prediction for packing fractions above $\sim \, 30\%$ coincides with the use of the close random packing algorithm^41^, which is necessary to generate ensembles beyond the random packing limit ($\sim \, 38\%$) efficiently. In dense packing, the local order between the micro-particles influences the small-angle scattering. In particular, the structure near the wall of the cubic sample container exhibits partial ordering since the boundary condition will result in sphere locations in direct contact with the wall surface. A generalized quantitative interpretation of the impact of the packing fraction on the dark-field signal has been given by Gkoumas et al.^42^. Overall, the dark-field signal is highly dependent on the structure of the sample. This can be seen especially from the distinct behavior of the dark-field signal at structure sizes comparable to the autocorrelation distance, as in grating-based systems^21,37^. The sub-linear dependence of the dark-field signal on the sample thickness reported in the experimental study of Vittoria et al.^43^ has not been observed in our case. Therefore, this dependence may be due to beam hardening or the speckle tracking algorithm. In addition, the wave-optics simulation does not consider the contributions of inelastic scattering, such as Compton scattering, since it uses a linear superposition of simulations performed for strictly monochromatic wave-fields^26^. To consider the contribution of inelastic scattering, which can be non-negligible for thicker samples than considered in this work, the wave-optics simulations can be complemented by Monte-Carlo simulations^44^.

While the dark-field signal strength increases when the speckle size approaches the scattering-associated length scale, speckle-based X-ray imaging requires that the speckle pattern is directly resolved by the detector system. This can be achieved for small speckle sizes with conventional high-efficiency flat-panel detectors with a pixel size of 50–200 $\upmu \hbox {m}$ by exploiting the geometric magnification of the X-ray source^16^. For pre-clinical studies that do not require a large field-of-view, detectors with small pixels are sufficient for dark-field imaging. Although a small speckle size is favorable for the dark-field signal strength, it could limit the performance of conventional speckle displacement retrieval algorithms^45^. While this was not relevant for the sample design in this work, it is of major importance for speckle-based X-ray imaging in general. From the good agreement between the simulation results and the theoretical dark-field prediction in Fig. 4A, it can also been concluded that speckle size does not change upon propagation within the near-field^18^.

From the good agreement between the simulated and theoretical dark-field values in Fig. 5A, it can be seen that the size of X-ray speckles within the near-field regime was independent of the X-ray beam energy. While the transmission signal follows the expected behavior according to $\beta \propto E^{-4}$, the energy dependence of the dark-field signal depends on the autocorrelation distance. Based on Eq. (5) and $\delta \propto E^{-2}$, the dark-field extinction coefficient for an ensemble of spheres is proportional to $E^{-2}$ if $d\le \xi$ and can vary up to $E^{-4}$. Changes in the dark-field signal for polychromatic spectra have also been observed for grating-based systems^46^ and were explained by a beam-hardening-induced increase or decrease in visibility, which in turn alters the dark-field signal. As the transmission is reduced (i.e., for increasing lead thickness), the mean energy of the transmitted photons increases. The visibility of the speckle pattern peaks at X-ray energies of about 17 keV^24^, which is different from grating interferometers where the visibility spectrum has a more complex shape with the peak visibility located near the design energy. Hence, beam-hardening causes a reduction of the speckle pattern visibility ($v_{{\text {sam}}}$). Since the visibility of the reference scan (i.e., without lead and sample; $v_{{\text {ref}}}$) remains unchanged, a lower dark-field signal is measured for the same scattering sample (cf. Eq. (4)). This effect can be seen for the PMMA sample in Fig. 5B. In addition, the increased mean energy also directly affects the dark-field extinction coefficient (Fig. 5A) and thereby causes an increase of the dark-field signal. This can be seen as a hardening of the visibility spectrum downstream the sample similar to conventional beam-hardening^46^. The effect was larger for samples with lower absolute dark-field signal. For decreasing transmission, this effect can partially or even fully compensate the aforementioned beam-hardening induced visibility change as it was observed for the SiO$_2$ sample in Fig. 5B.

Speckle-based imaging can be treated as a generalization of single-grating X-ray imaging. It allows multi-contrast imaging with a substantially simpler, cheaper, and more dose-effective setup, and does not suffer from phase-wrapping effects. However, since both speckle size and propagation distance depend on the geometric magnification, the autocorrelation distance will be independent of the magnification. Hence, for typical speckle sizes required with current conventional flat-panel detectors, the sensitivity will be lower than that of Talbot grating interferometers, where the period of the analyzer grating can be as small as a few micrometers. Further development and extension of speckle-based imaging are required to enhance this performance, potentially by developing a hybrid system of the two.

In this work, we only focused on dark-field and transmission signals due to the particular sample design; however, in general, speckle displacement might also occur due to extended phase-gradients. While current digital tracking algorithms can obtain all signals simultaneously, theoretical studies have shown that there can be cross-talk between the various signals^47^. To what extent X-ray interactions with heterogeneous anatomy influence the dark-field signal extraction needs to be investigated (e.g., for lung imaging the scattering caused by the rib cage and spine). A disadvantage of the single image mode (i.e., only one reference and one sample image) is that the speckle size (or more precisely the subset size used for the signal extraction) limits the spatial resolution. This can be overcome by using extensions of the original scenario by acquiring multiple images of the diffuser at different transverse positions^25^, which can be seen as equivalent to phase stepping in grating interferometry^48^. It should be noted that the speckles still need to be fully resolved.

The investigated samples were based on ensembles of mono-disperse spheres, which cannot be used to accurately model the dark-field signal generated from realistic biological tissue. However, the results reveal the important impact of the sample’s structural length scale and the speckle size on the dark-field signal, which is highly relevant for future biomedical applications. In addition, the precisely controllable sample parameters provide an ideal scenario for quantitatively investigating the dark-field signal in speckle-based X-ray imaging. Micro-sphere suspensions can be also imaged experimentally^36,37^, which will be useful for validation in future work.

The enhanced sensitivity of the dark-field signal over the conventional transmission signal for diagnosing structural variations of lung tissue has been demonstrated in pre-clinical and clinical in vivo studies using a Talbot–Lau grating interferometer^49,50^. Nevertheless, speckle-based dark-field imaging would allow a much simpler and cheaper setup, with essentially no field-of-view restrictions and with excellent dose-efficiency. This makes it a promising candidate for diagnostic pre-clinical lung imaging and further development could enable a possible pathway towards clinical applications.
